# Selectivity of Cobalt Corrole for CO vs. O_2_ and N_2_ in Indoor Pollution

**DOI:** 10.1038/s41598-017-15228-5

**Published:** 2017-11-06

**Authors:** Xia Sheng, Hailiang Zhao, Lin Du

**Affiliations:** 1 College of Chemistry, Chemical and Environmental Engineering, Henan University of Technology, Lianhua Street 100, 450001 Zhengzhou, China; 2 Environment Research Institute, Shandong University, Shanda South Road 27, 250100 Jinan, China

## Abstract

Coal combustion causes indoor pollution of CO. In this work, DFT calculations on cobalt corrole (Co(Cor)) with three most common indoor gas molecules (N_2_, O_2_ and CO) were performed. The Mulliken spin densities show that the ground states of Co(N_2_)(Cor), Co(CO)(Cor) and Co(OC)(Cor) have an anti-ferromagnetic coupling fashion of the electrons on the Co 3*d*_*z*_^2^ orbital and the π orbital of the corrole ring. However, Co(O_2_)(Cor) has a triplet ground state. With the spin contamination corrections, the Co(N_2_)(Cor) binding energy was obtained at −50.6 kcal mol^−1^ (B3LYP-D3). While CO can interact with Co(Cor) in two different ways, and their binding energies were −22.8 and −10.9 kcal mol^−1^ (B3LYP-D3) for Co(CO)(Cor) and Co(OC)(Cor), respectively. The natural bond orbital charges on the axial ligands (NO, CO, OC) are increased upon the chemical bond formation. These are the cause of the shorten metal-ligand bond and the increase of the wavenumber of the metal-ligand bond vibrational transitions. While the charges for O_2_ are decreased, leading to bond elongation as well as the decrease of the wavenumber upon complexation. Overall, O_2_ was found to be hardly coordinated with Co(Cor). This study provides a detailed molecular understanding of interactions between a gas sensor and gaseous indoor air-pollutants.

## Introduction

Biomass fuel smoke is a major health risk in household air pollution (HAP), particularly in developing countries^1^. Carbon monoxide (CO) is colorless, odorless and tasteless, but highly toxic. CO poisoning is the most common type of fatal air poisoning in many countries^2^. It can be combined with hemogblobin (Hb) to produce a stable complex in red blood cells, namely, carboxyhemoglobin (COHb). After binding with hemoglobin, CO cannot be released as easily as oxygen (O_2_). As a result, Hb is ineffective for delivering O_2_ to bodily tissues. Consequently, several symptoms of CO poisoning may lead to headache, nausea vomiting, dizziness, fatigue and a feeling of weakness. One early study has shown that coal combustion mainly causes indoor pollution of CO with concentrations exceed 125 mg m^−3^ in the selected 10 coal-burning residences of Jilin province^3^. Thus, a CO detector is often used to detect the presence of CO gas in order to prevent CO poisoning.

Corroles have been received increasing attention in the past few years^4–13^. The reason for the interest in these porphyrin-related macrocycles is mainly due to the coordination chemistry of corroles, which show unique and intriguing behaviors that are clearly distinguishable from those of porphyrins. Just like porphyrins, corroles are 18-electron aromatic macrocycles as well, except that they have a direct link between two pyrrole rings and, when fully deprotonated, are trianionic ligands. Its framework may be regarded as an aromatic analogue of corrin, a unit of Vitamin B12 coenzyme. The formal oxidation state of the coordinated metal in corrole derivatives is one charge higher than that of the corresponding metalloporphyrinates^4^. Corroles are trianionic ligands which can stabilize metal ions in their high-valent oxidation states^5^. Moreover, in contrast to the closely related porphyrin-based systems, corrole tends to be involved as a non-innocent ligand, forming π-radical species^6^. In this particular study of cobalt, porphyrins stabilize the (+II) oxidation state while cobalt(III) corroles are obtained. Corroles have been studied for applications in several fields. Cobalt corroles become one of the most studied corrole complexes after new synthetic methods for corroles were developed in recent 20 years by different independent groups. The four-coordinate cobalt corrole system was characterized as an intermediate-spin Co(III) (*S* = 1) center bonded with a corrole^3−^ (Cor^3−^) ligand in a square planar coordination environment in both experimental and theoretical studies^7^. When it transfers one unpaired electron from the paramagnetic Co(III) ion to the π orbitals of the corrole ligand, an electronic configuration of $({{\rm{d}}}_{{{\rm{x}}}^{2}-{{\rm{y}}}^{2}})$ ^2^ $({{\rm{d}}}_{{\rm{xz}}},\,{{\rm{d}}}_{{\rm{yz}}})$ ^2^ $({{\rm{d}}}_{{{\rm{z}}}^{2}})$ ^2^ was proposed^7^. The system tends to bind various axial ligands, such as triphenylphospine (PPh_3_), isonitrile (NO), phenyl (Ph), pyridine (Py), and amine^7–11^. The coordination property of Co(III)(Cor) causes difference from porphyrins. For example, Co(III) corroles are able to bind many donor molecules, while Co(II) porphyins have been shown also some capabilities to coordinate with CO, NO and O_2_, but cannot be used for gas sensing purposes as the former derivatives. Recently several papers have demonstrated that Co(III)(Cor) exhibited an infinite selectivity for CO with respect to N_2_ and O_2_^12,13^. This specific property enables them to be used as sensing components for gas detectors from the selective coordination of CO to the central Co(III) ion by a chemisorption process where N_2_ and O_2_ hardly coordinates.

We undertook a quantum chemical study of Co(Cor) interactions with diatomic gas donor molecules (O_2_, N_2_ and CO) from a molecular level. Quantum theory of atoms in molecules (AIM) and natural bond orbital (NBO) analysis was performed to understand the nature of weak interaction in the five-coordinate cobalt corrole complexes. This work is imperative to understand the nature of the adsorption bonding interactions.

## Results and Discussion

In this study, we reported investigations of the molecular interactions of Co(Cor) with a series of common atmospheric diatomic ligands by different density functional methods. The unsubstituted corrole ligand was used for the theoretical calculations. The optimized structures of the Co(L)(Cor) (L = O_2_, N_2_, CO, OC) obtained at the BP86/def2-TZVP level are provided in Figures S1-S4 (Supporting Information). Only the most stable structures are discussed in the paper. The important parameters affecting indoor air pollution from a molecular level by analyzing the results of AIM, NBO as well as the binding energies were given in details.

### Electronic configurations and spin densities

Previous study on Co(Cor) indicates the ground state is a Co(III) triplet ground state with an electronic configuration of $({{\rm{d}}}_{{{\rm{x}}}^{2}-{{\rm{y}}}^{2}})$ ^2^ $({{\rm{d}}}_{{\rm{xz}}})$ ^↑^ $({{\rm{d}}}_{{\rm{yz}}})$ ^↑^ $({{\rm{d}}}_{{{\rm{z}}}^{2}})$ ^2^ ^14^. The ground states of both the CO and the N_2_ free gases are singlet states with closed-shell electronic configurations, but for the O_2_ free gas the ground state is a triplet state with two unpaired electrons occupying two degenerate anti-bonding molecular orbitals. Upon complexation with the ligands, the spin state of the central cobalt atom is changed from an intermediate-spin (*S* = 1) state to a low-spin (*S* = 0) state. The Co(Cor) system has *C*_*2v*_ symmetry, and the five-coordinate Co(L)(Cor) systems possess either *C*_*s*_ or *C*_1_ symmetry. In this study, the Co-L bond distances were predicted much large by B3LYP-D3/def2-TZVP. In some cases, the bond was unexpected broken, for instance, the Co-O_2_ bond distance in the triplet state was predicted at 3.250 Å (B3LYP-D3/def2-TZVP), and the O-O distance (1.205 Å, B3LYP-D3/def2-TZVP) is also close to the free O_2_ value (1.204 Å, B3LYP-D3/def2-TZVP). This implies that the B3LYP-D3 functional is not suitable in predicting the structures for this type of weak interaction. On the other hand, the Co-L bonds predicted by BP86/def2-TZVP looks much reasonable, i.e., 1.727–1.902 Å for the Co-CO distances; 1.964 Å for the Co-OC distance; 1.788–2.106 Å for the Co-N_2_ distances; 1.802–1.854 Å for the Co-O_2_ distances. Furthermore, the single point calculations with the B3LYP/def2-TZVP, OLYP/def2-TZVP, B3LYP-D3/def2-TZVP levels of theory were carried out, making use of the BP86/def2-TZVP optimized structures. Since, CO has two binding sites: the oxygen atom and the carbon atom. Both of the two possible binding sites were considered in this study.

The corrole ligand belongs to the point group C_2v_. The two HOMO corrolate π orbitals are near-degenerate, which are represented by either a_2_ or b_2_ symmetry. Similarly, the two LUMO corrolate π orbitals are represented by either a_2_* or b_2_* symmetry. The a_2_ and b_2_ HOMOs of corrole are close analogues of the a_1u_ and a_2u_ porphyrin HOMOs, respectively. They are known as the Gouterman’s four-orbital to explain the electronic absorption spectra of porphyrins^15^. Co(Cor) tends to bind various axial ligands, thus the electrons in the HOMO might be readily excited into the cobalt 3d orbitals to form a Co(II) π-cation radical state. The Mulliken spin densities of the Co(L)(Cor) singlet and triplet states are presented in Tables 1 and 2. A quantity that is useful in this respect is the so-called effective number of unpaired electrons (NUE) and denoted as^16,17^:

1 $${\rm{NUE}}=\sum _{i=1}^{nat.\,orbs}{n}_{i}(2\,-\,{n}_{i})=2( < {S}^{2} > -{S}^{2})$$

where n_i_ (0 ≤ n_i_ ≤ 2) is the occupation number of the natural orbital; $< {S}^{2} > \,$is the expectation value of the total spin-squared operator; *S* is the total spin quantum number. NUE may be used to diagnose the character of the DFT solution obtained for any open-shell state.

**Table 1:** Mulliken spin densities (a.u.), expectation values <S^2^> and binding energies (*BEs*, kcal mol^−1^) for the Co(L)(Cor) singlet states.

Methods	<S^2^>	Co	Cor	L	NUE^a^	*BE* ^b^
**Co(O**_**2**_ **)(Cor)**						
BP86	0.0	0.0	0.0	0.0	0	−7.7
OLYP	0.0	0.0	0.0	0.0	0	8.6
B3LYP	0.0	0.0	0.0	0.0	0	29.4
B3LYP^c^	1.3247	−0.5162	−0.8813	1.3975	2.6	10.6 (13.1)
B3LYP-D3	0.0	0.0	0.0	0.0	0	24.9
B3LYP-D3^c^	1.3247	−0.5162	−0.8813	1.3975	2.6	6.1 (9.0)
**Co(N**_**2**_ **)(Cor)**						
BP86	0.0	0.0	0.0	0.0	0	−41.5
OLYP	0.0	0.0	0.0	0.0	0	−29.7
B3LYP	0.0	0.0	0.0	0.0	0	−30.3
B3LYP^c^	0.7074	0.8026	−0.8345	0.0319	1.4	−45.9 (−35.0)
B3LYP-D3	0.0	0.0	0.0	0.0	0	−35.0
B3LYP-D3^c^	0.7074	0.8026	−0.8345	0.0319	1.4	−50.6 (−39.7)
**Co(CO)(Cor)**						
BP86	0.0	0.0	0.0	0.0	0	−34.1
OLYP	0.0	0.0	0.0	0.0	0	−21.4
B3LYP	0.0	0.0	0.0	0.0	0	−14.1
B3LYP^c^	0.2105	0.4085	−0.4294	0.0209	0.4	−18.3 (−14.3)
B3LYP-D3	0.0	0.0	0.0	0.0	0	−18.5
B3LYP-D3^c^	0.2105	0.4085	−0.4294	0.0209	0.4	−22.8 (−18.8)
**Co(OC)(Cor)**						
BP86	0.0	0.0	0.0	0.0	0	6.4
OLYP	0.0	0.0	0.0	0.0	0	17.1
B3LYP	0.0	0.0	0.0	0.0	0	15.8
B3LYP^c^	0.9070	0.9882	−0.9922	0.0040	1.8	−6.5 (3.7)
B3LYP-D3	0.0	0.0	0.0	0.0	0	11.5
B3LYP-D3^c^	0.9070	0.9882	−0.9922	0.0040	1.8	−10.9 (−0.7)

^a^NUE = *effective number of unpaired electrons*. ^b^ *BEs* corrected with ZPVE. ^c^Broken-symmetry (BS) solution. Values corrected for spin contamination, uncorrected values given within parentheses.

**Table 2:** Mulliken spin densities (a.u.), expectation values <S^2^> and binding energies (*BEs*, kcal mol^−1^) for the Co(L)(Cor) triplet states.

Methods	< S^2^>	Co	Cor	L	NUE^a^	*BE* ^b^
**Co(O**_**2**_ **)(Cor)**						
BP86	2.0730	−0.0213	0.8653	1.1560	2.1	−9.7
OLYP	2.1133	−0.1233	0.8862	1.2371	2.2	4.5
B3LYP	2.4304	−0.5583	1.0158	1.5425	2.8	3.1
B3LYP-D3	2.4304	−0.5583	1.0158	1.5425	2.8	−1.3
**Co(N**_**2**_ **)(Cor)**						
BP86	2.0157	1.2844	0.6941	0.0215	2.0	−36.9
OLYP	2.0235	1.3469	0.6401	0.0130	2.0	−28.1
B3LYP	2.0214	1.2042	0.7834	0.0124	2.0	−38.8
B3LYP-D3	2.0214	1.2042	0.7834	0.0124	2.0	−43.3
**Co(CO)(Cor)**						
BP86	2.0117	1.0083	0.8650	0.1267	2.0	−18.3
OLYP	2.0191	1.0675	0.8214	0.1111	2.0	−7.0
B3LYP	2.0183	0.9770	0.9390	0.0840	2.0	−8.7
B3LYP-D3	2.0183	0.9770	0.9390	0.0840	2.0	−12.9

^a^NUE = effective number of unpaired electrons. ^b^BEs corrected with ZPVE.

**Figure 1:**
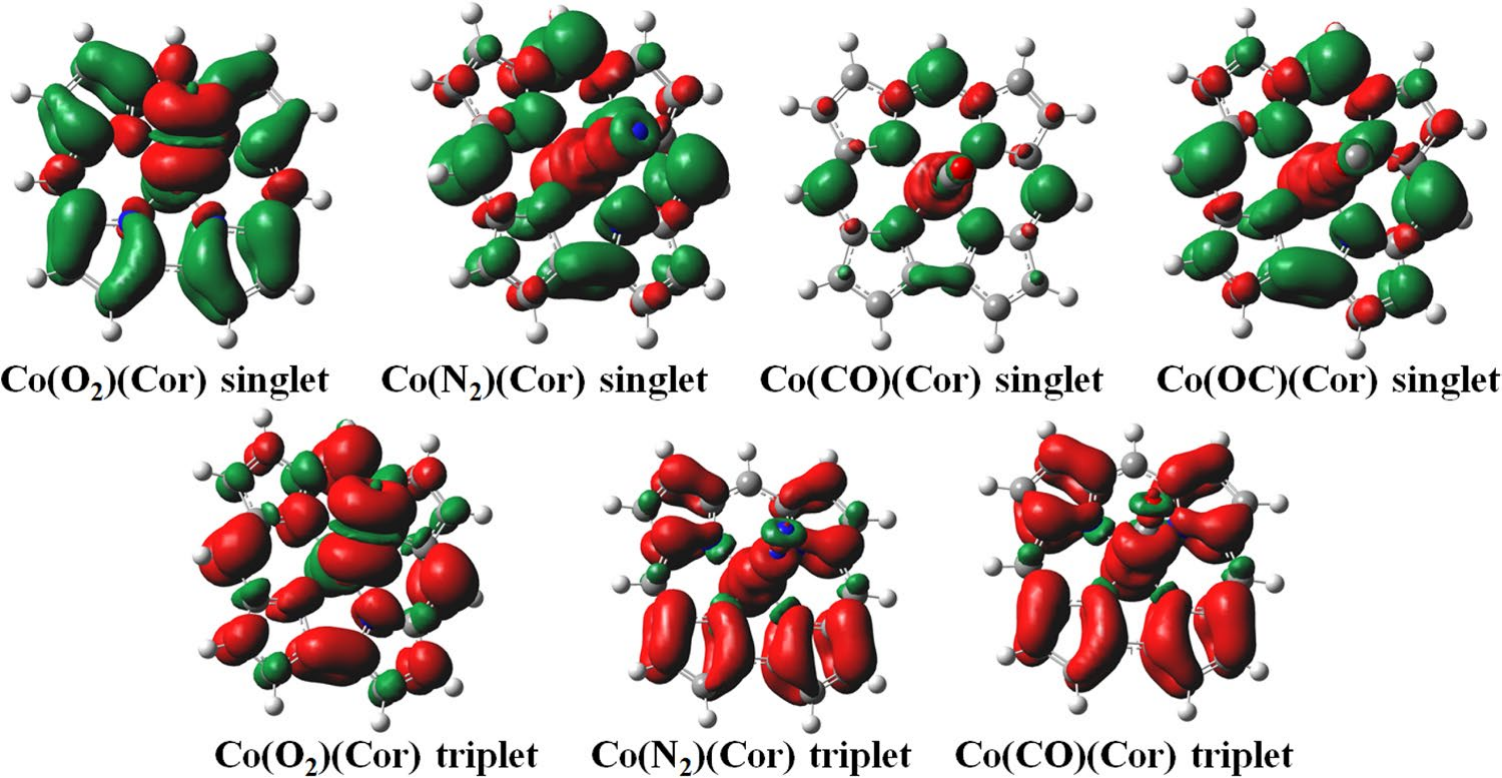
DFT spin density plots for the broken-symmetry singlet and triplet states of the Co(L)(Cor) (L = O_2_, N_2_, CO, OC) systems (spin density in red/green, contour value 1 × 10^−3^ a.u.).

As shown from Table 1, the two pure functionals, OLYP and BP86 obtained a restricted solution (<*S* ^2^>  = 0). It is due to the degree of separation of the positive and negative spin densities, which depends on the *xc* functional. The separation by pure functionals is relatively small^18^. By mixing HF with DFT, the hybrid functionals, B3LYP (20% HF) and B3LYP-D3 (20% HF) obtain a Broken Symmetry (BS) solution. The two pure functionals predict the closed-shell singlet state for the Co(L)(Cor) (L = O_2_, N_2_, CO, OC), which are non-broken symmetry solutions and can be described as low-spin Co(III)-like system. Contrary to this expectation, the hybrid functionals show that the most stable state is a BS solution. For the Co(L)(Cor) (L = N_2_, CO, OC) BS states, the electron in the π orbital (“a_2u_” HOMO) in the corrole HOMO is anti-ferromagnetically coupled with the unpaired electron in the $3{d}_{{z}^{2}}$ orbital. The Co ion in the complex is better described as a partial Co(II) character and the corrole ring is a radical with a charge of “−2”^19^. In the BS singlet Co(II)(*S* = 1/2)(L)(Cor^•2−^), the negative Mulliken spin populations at the *meso* carbons (Fig. 1) further proves the biradical character of the singlet system. As seen from the spin density plot (Fig. 1), the unpaired electron in the Co $3{d}_{{z}^{2}}\,$orbital is anti-ferromagnetically coupled with another unpaired electron in the π orbital on the corrole ring to form a singlet state.

For the Co(O_2_)(Cor) BS state (B3LYP-D3/def2-TZVP), the spin population of O_2_ is 1.3975. In contrast, the free O_2_ gas molecule has two unpaired electrons. There is no significant bonding between O_2_ and Co(Cor). The electronic configuration for the Co(Cor) fragment is described as one electron in the metal anti-ferromagentically coupled one electron in the corrole. The expectation value, 1.3247, also shows that there is a large spin contamination. Because it is a Cor^2−^ π radical state with electron transfer from corrole π orbital to one of the metal orbitals. The ground state was found to be the triplet state, which is the same as BP86. All of the above models are different from the models for iron porphyrins: Weiss model where Fe(III) is anti-ferromagnetically coupled with O_2_^−^ to form a singlet ground state and Pauling model where the system was described as Fe(II)(*S* = 0)O_2_(*S* = 0)^20^.

The BS singlet state contains some amount of triplet, quintet, …, states. The contamination was approximately corrected by the standard spin projection technique (Eqs 2–4)^21,22^. The BP86 and OLYP functionals could not find the BS solution. The correction to the energy was done for B3LYP and B3LYP-D3, and shown in Table 3. From the expectation values, we can notice that the BS singlet states are strongly contaminated by the triplet state, for Co(O_2_)(Cor) it contains 34% singlet and 66% triplet character for Co(O_2_)(Cor). For the other three system, the triplet character is much smaller, while 65% singlet and 35% triplet character for Co(N_2_)(Cor), 89% singlet and 11% triplet character for Co(CO)(Cor), and 55% singlet and 45% triplet character for Co(OC)(Cor). Meanwhile, the correction for Co(O_2_)(Cor) is positive, for other three systems are negative. This is due to the different ground spin state as shown in Table 4, where it shows the relative energies of the singlet and triplet electronic states of Co(L)(Cor) (L = O_2_, N_2_, CO, OC) with respect to the ground state. The results obtained with OLYP and B3LYP are listed in the supporting information (Table S1). As seen from Table 4, the pure functional delivers singlet ground states for Co(N_2_)(Cor), Co(CO)(Cor) and Co(OC)(Cor), but a triplet ground state for Co(O_2_)(Cor). While, B3LYP-D3 gives a different ground state for Co(N_2_)(Cor). However, the singlet state of Co(N_2_)(Cor) becomes the ground state after the spin contamination corrections. The singlet-triplet splitting is relatively different to the method and also the systems. The BP86 methods give a 1.1, −6.3, and −17.5 kcal mol^−1^ for Co(O_2_)(Cor), Co(N_2_)(Cor) and Co(CO)(Cor), respectively. In contrast, the B3LYP-D3 method obtained relatively different singlet-triplet splitting energies, where the corresponding data for the three systems are 8.2, −6.5, and −9.8 kcal mol^−1^. However, the trends observed between them are the pure functional overstabilized low-spin relative to high-spin. In contrast, the high-spin state becomes more favored over the low-spin state with hybrid functionals.

**Table 3:** Spin projection results (kcal mol^−1^) for the singlet *BS* states of Co(L)(Cor) (L = O_2_, N_2_, CO, OC).

System	Δ*E*_BS-HS_	<*S* ^2^>_BS_	<*S* ^2^>_*HS*_	J	Δ*E*_*J*_
Co(O_2_)(Cor)	1.4	1.3247	2.3653	1.3	1.8
Co(N_2_)(Cor)	−12.1	0.7074	2.0220	−9.2	−6.5
Co(CO)(Cor)	−18.7	0.2105	2.0201	−10.3	−2.2
Co(OC)(Cor)	−7.7	0.9070	2.0365	−6.8	−6.2

**Table 4:** Relative energies (kcal mol^−1^) of the singlet and triplet electronic states of Co(L)(Cor) (L = O_2_, N_2_, CO, OC) with respect to their corresponding ground state.

State	Configuration	O_2_	N_2_	CO	OC
**BP86/def2-TZVP**					
Singlet	$({{\rm{d}}}_{{{\rm{x}}}^{2}-{{\rm{y}}}^{2}})$ ^2^ $({{\rm{d}}}_{{\rm{xz}}})$ ^2^ $({{\rm{d}}}_{{\rm{yz}}})$ ^2^ $({{\rm{d}}}_{{{\rm{z}}}^{2}})$ ^0^ ${({\rm{Cor}})}^{2}$	1.1	0.0	0.0	0.0
Triplet	$({{\rm{d}}}_{{{\rm{x}}}^{2}-{{\rm{y}}}^{2}})$ ^2^ $({{\rm{d}}}_{{\rm{xz}}})$ ^2^ $({{\rm{d}}}_{{\rm{yz}}})$ ^2^ $({{\rm{d}}}_{{{\rm{z}}}^{2}})$ ^↑^ ${({\rm{Cor}})}^{\uparrow }$	0.0	6.3	17.5	NP
**B3LYP-D3/def2-TZVP**					
Singlet (BS)	$({{\rm{d}}}_{{{\rm{x}}}^{2}-{{\rm{y}}}^{2}})$ ^2^ $({{\rm{d}}}_{{\rm{xz}}})$ ^2^ $({{\rm{d}}}_{{\rm{yz}}})$ ^2^ $({{\rm{d}}}_{{{\rm{z}}}^{2}})$ ^↑^ ${({\rm{Cor}})}^{\downarrow }$	8.2 (6.4)	0.0 (0.0)	0.0 (0.0)	0.0 (0.0)
Singlet	$({{\rm{d}}}_{{{\rm{x}}}^{2}-{{\rm{y}}}^{2}})$ ^2^ $({{\rm{d}}}_{{\rm{xz}}})$ ^2^ $({{\rm{d}}}_{{\rm{yz}}})$ ^2^ $({{\rm{d}}}_{{{\rm{z}}}^{2}})$ ^0^ ${({\rm{Cor}})}^{2}$	25.2 (25.2)	13.2 (4.7)	2.4 (0.2)	18.3 (12.1)
Triplet	$({{\rm{d}}}_{{{\rm{x}}}^{2}-{{\rm{y}}}^{2}})$ ^2^ $({{\rm{d}}}_{{\rm{xz}}})$ ^2^ $({{\rm{d}}}_{{\rm{yz}}})$ ^2^ $({{\rm{d}}}_{{{\rm{z}}}^{2}})$ ^↑^ ${({\rm{Cor}})}^{\uparrow }$	0.0 (0.0)	6.5 (−2.0)	9.8 (7.6)	NP

^a^NP = not possible. ^a^Values corrected for spin contamination, uncorrected values given within parentheses.

### Geometric parameters

In Table 5 the mean bond lengths, cobalt out-of-plane displacement and ligand stretching frequency for the selected systems are given, together with the ligand bond length and the ligand stretching frequencies of the free gases. The mean bond lengths of Co-N in the four systems are very close to each other. The axil ligand bond lengths and stretching frequencies were slightly larger than the ones in free gas phase. Large deviation in bond lengths was found in the Co-L bond length, while 1.802/1.854 Å for Co(O_2_)(Cor), 1.788/2.106 Å for Co(N_2_)(Cor), 1.727/1.902 Å for Co(CO)(Cor) and 1.964 Å for Co(OC)(Cor). This is due to the difference ionic radius of the gas molecules.

**Table 5:** Selected bond lengths (Å), cobalt out-of-plane displacements (Å), angles (degrees) and ligand stretching frequencies (cm^−1^) for the lowest Co(L)(Cor) states and the free gas molecules at the BP86/def2-TZVP level^a^.

parameters	Co(O_2_)(Cor)		Co(N_2_)(Cor)		Co(CO)(Cor)		Co(OC)(Cor)
	Singlet	Triplet	Singlet	Triplet	Singlet	Triplet	Singlet
r_(Co-N)_	1.889	1.894	1.897	1.886	1.895	1.898	1.893
r_(Co-L)_	1.802	1.854	1.788	2.106	1.727	1.902	1.964
θ_(Co-L)_	121.4	120.4	180.0	168.7	180.0	164.4	180.0
d_(Co-N4)_	0.301	0.223	0.316	0.209	0.436	0.287	0.273
r_(L)_	1.264	1.268	1.119	1.108	1.154	1.150	1.149
${\rm{\upsilon }}$_(L)_	1256	1250	2186	2263	2029	1994	1990
**free gas**							
r_(L)_	1.221		1.291		1.214		1.214
${\rm{\upsilon }}$_(L)_	1542		1480		1728		1728

^a^r_(Co-N)_ is the mean bond length between Co and the four nitrogen atoms. r_(Co-L)_ is the distance between Co and the ligand. θ_(Co-L)_ is the angle between Co and the ligand. d_(Co-N4)_ is the cobalt out-of-plane (plane is defined by the four nitrogen atoms) displacement. r_(L)_ is the bond length of the ligand. ${\rm{\upsilon }}$_(L)_ is the ligand stretching vibrational frequency.

In a similar study, the Co-CO distance was calculated at 1.827/2.009 Å (B3LYP-D3/STO-TZ2P)^19^, slightly longer than the values in our study. Pure functionals always obtain a substantially shorter metal-ligand distance, which are in line with the similar studies^23^. The closest analogue is the binding with cobalt porphyrins. The CO interacts with Co(II) porphyrins show to have much weaker Co-CO bond distances at 2.01 Å which is also larger than the one studied here^24^. In Co(Cor) (without axil ligand), the cobalt out-of-plane displacement is calculated at 0.065 Å (BP86/def2-TZVP). After binding with the gas molecules, Co moves out towards the axial ligand. The Co out-of-plane displacements differs for the four systems: the Co(CO)(Cor) singlet state gives the largest displacement about 0.436 Å; in the other systems, the displacements are less than 0.32 Å. N_2_, CO and OC bind with Co(Cor) in a linear mode with σ-donation and π-back donation in the singlet states, but they are bonded in bent-modes with angles of 164.4°–168.7° in the triplet states. Meanwhile, O_2_ binds in a bent-mode with an angle of about 120.4°–121.4° in both the singlet and triplet states. The CO and O_2_ binding angles are in good agreement with the ones presented in the iron porphyrin histidine complex^25^. In the linear Co-L (L = N_2_, CO, OC) bond, the Co $3{d}_{{z}^{2}}\,\,$orbital is destabilized by the σ-donation from the axial ligand into the Co $3{d}_{{z}^{2}}$ orbital. Similarly, the Co 3 ${d}_{xy}$ orbital is destabilized by the σ-antibonding interactions with the four corrole N atoms. While, the ${{\rm{d}}}_{{{\rm{x}}}^{2}-{{\rm{y}}}^{2}}$ orbital is an essentially non-bonding orbital. In the triplet state, one of the electron from the bonding orbitals (Co $3{d}_{{z}^{2}}$ or 3 ${d}_{xy}$) excites into its corresponding σ-antibonding orbital. In the case of occupying the $3{d}_{{z}^{2}}$ antibonding orbital, the overlap of the Co $3{d}_{{z}^{2}}$ and the ligand σ orbital is much reduced. Thus, a bending of the Co-L bond is obtained^26^. The ligand bonds in the Co(L)(Cor) (L = N_2_, CO, OC) systems are much shorter than the ones in the free gases, due to the interactions between cobalt corrole and axial ligands. On the other hand, the Co-O_2_ bond is slightly increased in the Co(O_2_)(Cor) as compared with the O_2_ free gas. This in turn results in a red or blue shift of the ligand bond upon complexation. The C-O and N-N stretching frequencies are sharply increased by 262–783 cm^−1^ in the Co(L)(Cor) (L = N_2_, CO and OC) systems, but the O_2_ stretching frequency in Co(O_2_)(Cor) is decreased by 286–292 cm^−1^ with respect to the O_2_ gas molecule. The changes of the ligand stretching frequencies as well as the changes of the Co-L bond lengths indicate the strength of the molecular interactions: Co(N_2_)(Cor) > Co(CO)(Cor) > Co(OC)(Cor) > Co(O_2_)(Cor).

### Binding energies

The binding energy (interaction energy) is a most convincing measure of the strength of molecular interactions. The relative stability of similar complexes is in accordance with the calculated binding energies. For a stable complex, the value of *BE* is often negative. The larger the absolute value of *BE*, the stronger the strength of the corresponding molecular interactions. The calculated binding energies are collected in Tables 1–2. ZPVE obtained with BP86/def2-TZVP varies from about 1.0 to 3.7 kcal mol^−1^ depending on system, and they were included into all binding energies. In the previous study, the OLYP functional has been found to give a better match to the experimentally available binding energies for porphyrin systems than other functionals (PBE0, B3LYP, BP86)^20^. It clearly shows the DFT binding energies are strongly functionals dependent (Tables 1 and 2), which conforms with previous studies^14,20,26^. The strongest *BEs* are predicted by BP86, then the weakest *BEs* are obtained by B3LYP. This further proves the hybrid functionals are not suitable to calculate this type of weak interactions. On the other hand, as compared with the results obtained by OLYP, B3LYP-D3 gives a better match than BP86 and B3LYP. The energy differences between B3LYP and B3LYP-D3 are less than 5 kcal mol^−1^. This implies that the dispersion corrections are about 5 kcal mol^−1^. Thus, they are quite important in calculating binding energies. To the best of our knowledge, there is no experimental measurement on the binding energies for these type of corrole systems. The close study is the binding energy with CO and O_2_ to heme (porphyrin) system. In the computational study of the binding energies of CO and O_2_ to the four-coordinate iron porphyrin, the binding energies were calculated as −52.7 kcal mol^−1^ for CO and −43.6 kcal mol^−1^ for O_2_ (Fe: OLYP/QZVPP; ligand: OLYP/TZVPP)^20^. This is the same binding trend as our DFT results: CO forms a stronger bond than O_2_ does. Since N_2_ is isoelectronic with CO, the bonding in dinitrogen complexes is closely allied to that in carbonyl compounds, although N_2_ is a weaker *σ*-donor and *π*-acceptor than CO. However, we found that N_2_ forms a stronger bond than CO does. Moreover, the O_2_ hardly coordinates to Co(Cor). This is a different conclusion as the previous studies, where CO is a significantly selected by cobalt corrole with respect to N_2_ and O_2_^12,13^. The different conclusion is probably due to the selected system, where Barbe *et al*. synthesized six different corroles: 5,15-dimesityl-10-(4-aminophenyl) corrole, 5,15-dimesityl-10-(4-chloroacetamidophenyl) corrole, 5,15-dimesityl-10-(2,4,6-trimethoxyphenyl) corrole, 5,15-dimesityl-10-(4-acetamidophenyl) corrole, 5,15-dimesityl-10-(2,4,6-trimethoxyphenyl) corrole, and 5,10,15-tris(2,6-dichlorophenyl) corrole. In this study, a very simplified corrole was used. The groups on the *meso* carbons may modify the chemical properties of the corrole system.

### AIM and NBO analysis

**Figure 2:**
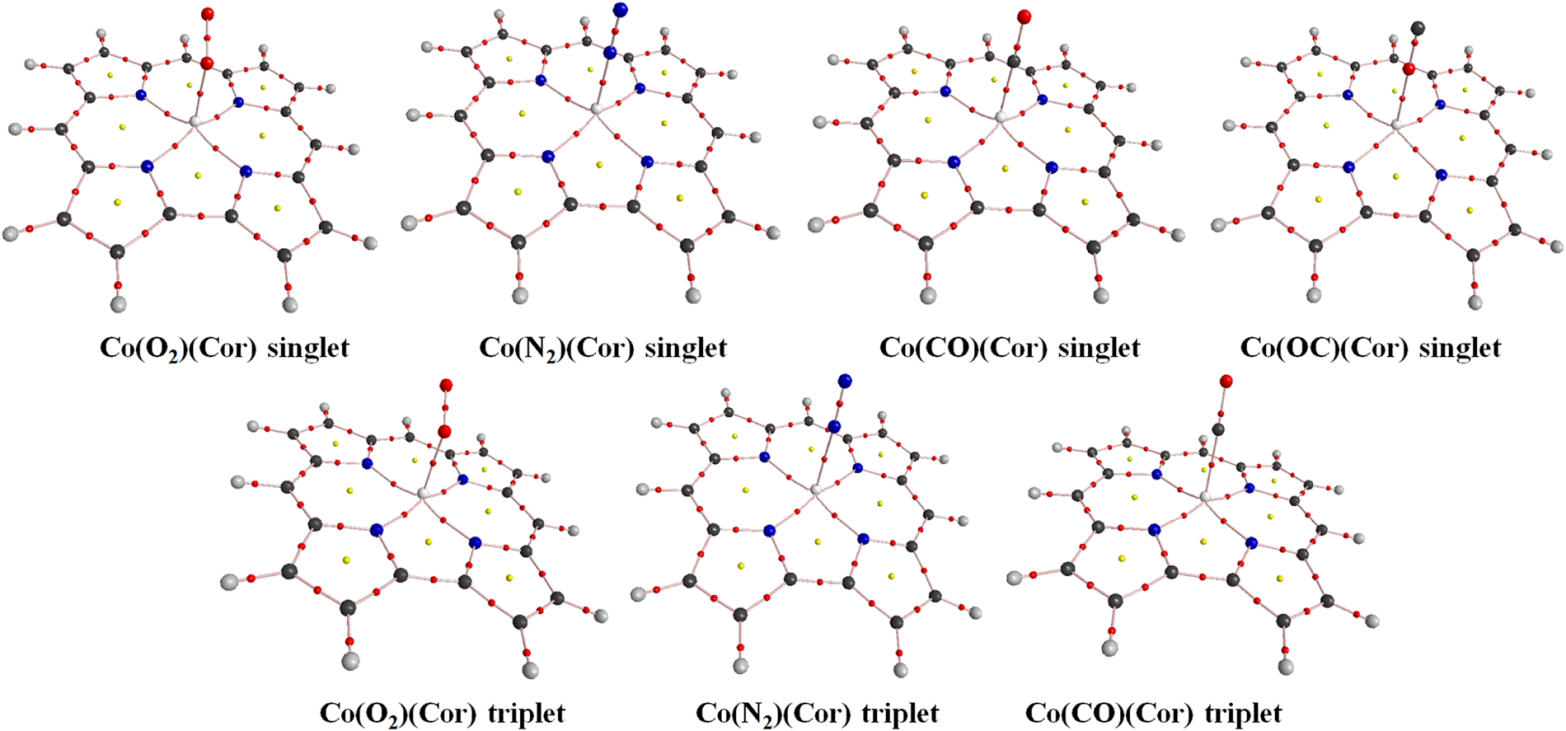
The AIM plots of the Co(L)(Cor) complexes obtained at the B3LYP-D3/def2-TZVP level. The bond critical points (BCPs) and ring critical points (RCPs) are presented by the *red* and *yellow* balls, respectively.

Topological analysis by using quantum theory of atoms in molecules (QTAIM) is commonly used to analyze structure, bonding and chemical reactivity. In this study, AIM analysis was used to evaluate the bond strength. AIM analysis was performed using the wavefunctions calculated at the B3LYP-D3/def2-TZVP level. The AIM plots of the complexes with bond critical points (BCPs), ring critical points (RCPs) and electron density paths are shown in Fig. 2. The AIM molecular graph shows the BCPs along the lines joining the Co atom and the ligand, which clearly prove the presence of chemical bonds. The topological parameters, including electron densities *ρ*(r), Laplacian ∇^2^ *ρ*(r) at the BCPs with the B3LYP-D3/def2-TZVP method are listed in Table 6. It shows that the values of *ρ*(r) at BCP are in the range of 0.0514–0.1246 a.u. for the Co(L)(Cor) complexes. The positive values of ∇^2^ *ρ*(BCP) imply a closed-shell interaction between two bonded atoms^27^. In addition, the values of ∇^2^ *ρ*(BCP) are all positive as well, ranging from 0.3216–1.1472 a.u. It is well known that the higher values of *ρ*(BCP) indicate stronger molecular interactions. Therefore, as the results in Table 6, the *ρ*(BCP) values of the Co(L)(Cor) (L = N_2_, CO) singlet states are greater than their corresponding values of the triplet states. For Co(O_2_)(Cor), the *ρ*(BCP) values of the singlet states are smaller than that of its triplet state. Overall, this is in agreement with the trend of the interaction energies. In contrast, the ∇^2^ *ρ*(BCP) values for all the Co(L)(Cor) triplet states are significantly lower than their corresponding singlet states. The values of ∇^2^ *ρ*(BCP) are in line with the length of a given Co-L bond: the greater ∇^2^ *ρ*(BCP), the shorter the bond. For the singlet states, the sequence is CO > N_2_ > O_2_ > OC. On the other hand, the trend is O_2_ > CO > N_2_ for the triplet states.

**Table 6:** AIM parameters and NBO charge changes of Co and L from different complexes to monomer obtained at the B3LYP-D3/def2-TZVP level.

Conformer	State	*ρ*(BCP)	∇^2^ *ρ*(BCP)	Δ*q*(Co)	Δ*q*(L)
Co(O_2_)(Cor)	Singlet (BS)	0.0953	0.9240	−0.2777	−0.1441
	Singlet	0.1126	0.5385	−0.2805	−0.0948
	Triplet	0.1168	0.4885	−0.2461	−0.0746
Co(N_2_)(Cor)	Singlet (BS)	0.1233	0.9321	−0.4484	0.1737
	Singlet	0.1133	0.9901	−0.4542	0.2465
	Triplet	0.0522	0.3216	−0.2525	0.1385
Co(CO)(Cor)	Singlet (BS)	0.1203	1.1472	−0.7314	0.4587
	Singlet	0.1246	1.0735	−0.7333	0.4860
	Triplet	0.1035	0.5017	−0.1969	0.1994
Co(OC)(Cor)	Singlet (BS)	0.0514	0.6175	−0.2388	0.0955
	Singlet	0.0620	0.5313	−0.2261	0.1481

Electron density redistribution always accompanies the formation of a chemical bond. NBO analysis provides an efficient method for studying inter-molecular interactions and a convenient basis for investigating charge transfer (CT) in a molecular system. The charge donating centers and the charge accepting centers are often the lone pairs of the acceptor and the anti-bonding orbitals of the donor, respectively. Values listed in Table 6 are the calculated charge changes of the Co atom and the axial ligand from complex to a monomer. Here we observe a charge change in the central cobalt atom and the axial ligand. The negative values of the charge change mean the Co d obtains more charge upon a metal-ligand bond formation. For Co(L)(Cor) (L = N_2_, CO, OC), Δ*q*(L) are positive. Thus, they show the increase of the wavenumbers of the ligand vibrational transitions with respect to the gas monomers. However, Δ*q*(L) are negative for Co(O_2_)(Cor). As a result, the wavenumbers of O_2_ are decreased upon complexation. For all the singlet states, the sequence for the absolute values of Δ*q*(Co) is the same as that of ∇^2^ *ρ*(BCP), while the sequence for the triplet states is opposite trend as that of ∇^2^ *ρ*(BCP).

## Conclusions

The main aim of this study was to investigate the parameters affecting indoor air pollution from a molecular level. Theoretical study of the molecular interactions of cobalt corrole with a series of common atmospheric diatomic ligands (O_2_, N_2_, and CO) was performed with several DFT methods (BP86, OLYP, B3LYP, B3LYP-D3). The accuracy of the theoretical methods was corrected with spin contaminations. For N_2_, CO and OC, the ground state is a singlet state, in which the unpaired electron in the Co center is anti-ferromagnetically coupled with the unpaired electron in the corrole π cation radical. The complexes are better described as Co(II)(*S* = 1/2)(L)(Cor^•2−^). However, for Co(O_2_)(Cor), the ground state is a triplet state. AIM analysis revealed that molecular interactions were present in the complexes, forming a chemical bond. NBO analysis clearly indicated the existence of charge transfer. The calculated binding energies show that N_2_ forms the strongest complex with Co(Cor), while O_2_ hardly coordinates with Co(Cor). The reason may be due to the simplified corrole in this study. In our study, the results provide a better understanding of the molecular interaction between a gas sensor and indoor pollutants.

## Methods

**Figure 3:**
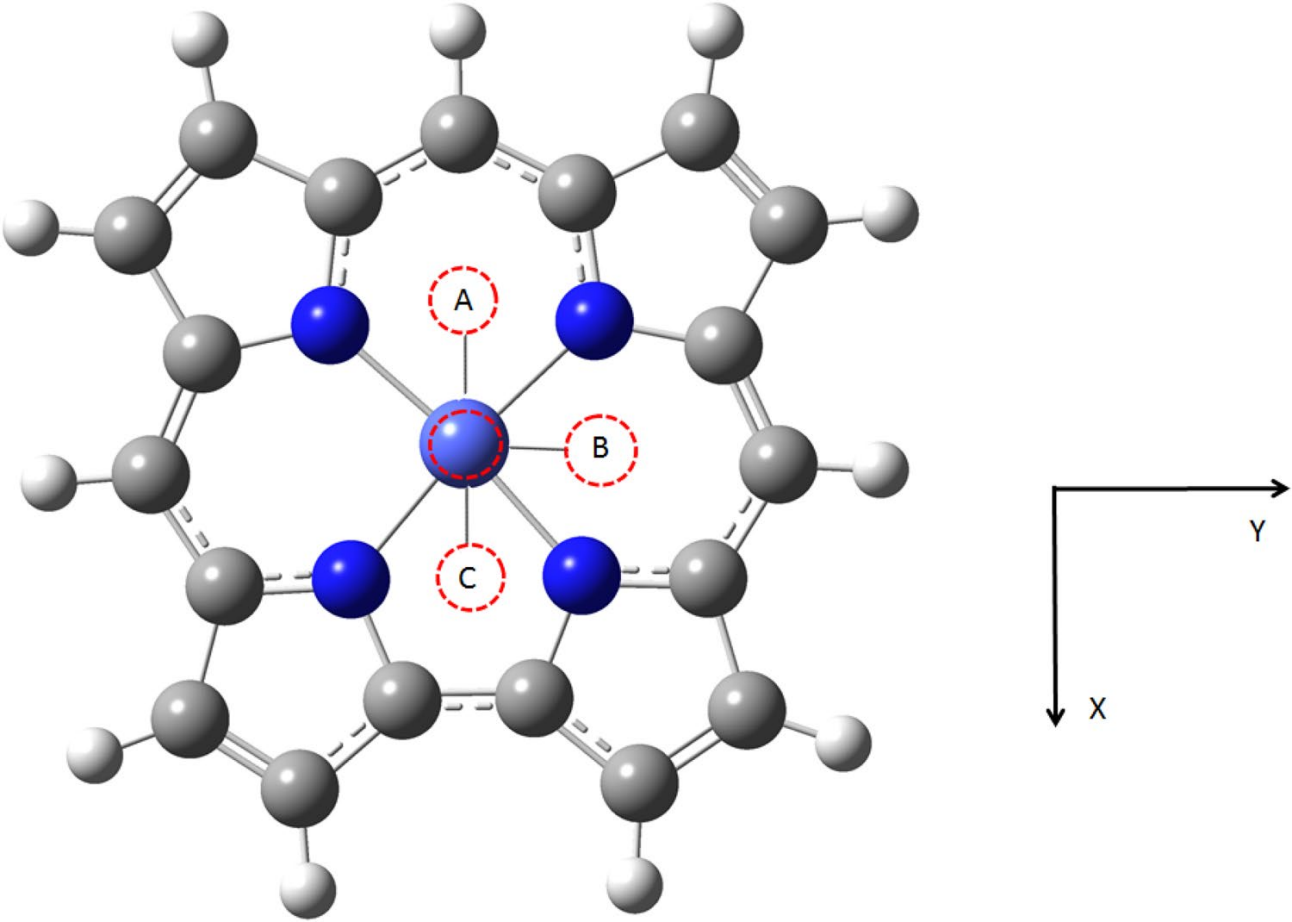
Molecular structure of Co(L)(Cor) (L = O_2_, N_2_, CO, OC). The ***A***, ***B*** and ***C*** positions are the possible ligand orientations upon complexation.

DFT calculations on Co(Cor) systems (Fig. 3) with several axial ligands, denoted as Co(L)(Cor) (L = O_2_, N_2_, CO, OC), were carried out with Gaussian 09 (Revision E.01) program package^28^. Geometries were optimized for all the possible low-lying spin states (Fig. 3, ligands at positions **A**, **B** and **C**) with BP86 plus the def2-TZVP basis set on all atoms. Co(Cor) system has a *C*_*2v*_ symmetry. All Co(L)(Cor) model systems possess either a *C*_*s*_ or *C*_1_ symmetry. In order to find the lowest-lying states, the *C*_1_ symmetry was used in all calculations. Frequency calculations to approximate zero-point vibrational energy (ZPVE) were performed at the BP86/def2-TZVP level. A “verytight” optimization convergence criteria and an “ultrafine” numerical integration grid were used for the DFT calculations to obtain accurate results^29,30^. Two more functionals were used as well. One hybrid functional was employed: B3LYP has 20% Hatree-Fock (HF) exchange and 80% Slater (S) exchange. Besides BP86, another pure Generalized Gradient Approximation (GGA) functional was used: OLYP. Moreover, B3LYP-D3 is a hybrid functional, containing Grimme’s D3 empirical dispersion correction^31^. The empirical dispersion correction has been shown to give an important contribution for general main group thermochemistry, kinetics, and noncovalent interaction^32^. The spin-unrestricted DFT formalism were used in all cases. For the (*S* = 0) state, both B3LYP and B3LYP-D3 give an unrestricted solution. For the two pure functionals, the unrestricted calculation converges to a restricted solution. The binding energies (*BEs*) are defined as the difference between the energies of the complex and the sum of the monomers, and then they are corrected with ZPVEs.

For the singlet states, a singlet biradical model (Broken Symmetry (BS) approach) was obtained with hydride functional, where there are two singly occupied molecular orbitals (SOMOs) of equal energy, and the spins of the two electrons are weakly anti-ferromagnetically coupled. However, the wavefunction is no longer an eigenfunction of the total spin, <*S* ^2^>, thus introducing some error. This error is called spin contamination. The contamination could be approximately corrected by a standard spin projection technique^21,22^. The correction to the energy of the BS state is

2 $$\Delta {E}_{J}=xJ$$

where *x* is measure of spin contamination in the BS state

3 $$x={\langle {S}^{2}\rangle }_{BS}\,-\,S(S+1)$$

while *J* is the effective exchange coupling constant of the Heisenberg-Dirac-van Vleck Hamiltionian^22,33,34^

4 $$J=\frac{{E}_{BS}\,-\,{E}_{HS}}{{\langle {S}^{2}\rangle }_{HS}\,-\,{\langle {S}^{2}\rangle }_{BS}}$$

where ${\langle {S}^{2}\rangle }_{HS}$ and ${\langle {S}^{2}\rangle }_{BS}$ are the expectation values of the total spin-squared operator for the two calculations, *S* is the total spin quantum number, *E*_*BS*_ is the energy of the BS state, *E*_*HS*_ is the energy of the high-spin (HS) state which is obtained from a separate energy calculation at the equilibrium geometry of the BS state.

The atoms in molecules (AIM) analyses were employed to predict the nature of the molecular interaction by searching the bond critical points (BCPs), ring critical points (RCPs) and calculating electron density ρ(r) and Laplacian ∇^2^ρ(r) at the BCPs. A detailed topological analysis was performed with the AIM2000 (version 2) program package^35,36^. Natural bond orbital (NBO) analysis was used to understand and describe the electron delocalization between the ligand and the metal center. The NBO program (version 3.1) as implemented in the Gaussian 09 package was implied to perform the NBO analysis.

## Electronic supplementary material

Supporting Information
