# Supplementary material for: Selectivity of Cobalt Corrole for CO vs. O2 and N2 in Indoor Pollution
Source: Sci Rep. 2017 Nov 6;7:14536. doi: 10.1038/s41598-017-15228-5 (PMC5674038; doi:10.1038/s41598-017-15228-5)
Supplement: Supplementary file 1 — Supporting Information [file 41598_2017_15228_MOESM1_ESM.pdf]

Supporting Information for

Selectivity of Cobalt Corrole for CO vs. O_2 and N_2 in Indoor Pollution

Xia Sheng,[1] Hailiang Zhao[1,2] and Lin Du[2,*]

[1] College of Chemistry, Chemical and Environmental Engineering, Henan University of Technology, Lianhua Street 100, 450001 Zhengzhou, China

[2] Environment Research Institute, Shandong University, Shanda South Road 27, 250100 Jinan, China

E-mail: lindu@sdu.edu.cn. Fax: 86-531-88366072. Phone: 86-531-88366072.

Table S1. Relative energies (kcal mol^{-1}) of the singlet and triplet electronic states of Co(L)(Cor) (L = O_2, N_2, CO, OC) with respect to their corresponding ground state [a]

State	Configuration	O_2	N_2	CO	OC
	PBE0/def2-TZVP				
Singlet	$(d_{x^2-y^2})^2(d_{xz})^2(d_{yz})^2(d_{z^2})^0(Cor)^2$	1.4	0.4	0.0	0.0
Triplet	$(d_{x^2-y^2})^2(d_{xz})^2(d_{yz})^2(d_{z^2})^{\uparrow}(Cor)^{\uparrow}$	0.0	0.0	0.3	NP
	OLYP/def2-TZVP				
Singlet	$(d_{x^2-y^2})^2(d_{xz})^2(d_{yz})^2(d_{z^2})^0(Cor)^2$	1.1	0.0	0.0	0.0
Triplet	$(d_{x^2-y^2})^2(d_{xz})^2(d_{yz})^2(d_{z^2})^{\uparrow}(Cor)^{\uparrow}$	0.0	3.2	16.1	NP
	B3LYP/def2-TZVP				
Singlet (BS)	$(d_{x^2-y^2})^2(d_{xz})^2(d_{yz})^2(d_{z^2})^{\uparrow}(Cor)^{\downarrow}$	8.2 (6.4)	0.0 (0.0)	0.0 (0.0)	0.0 (0.0)
Singlet	$(d_{x^2-y^2})^2(d_{xz})^2(d_{yz})^2(d_{z^2})^0(Cor)^2$	25.2 (25.2)	13.0 (4.5)	2.2 (0.0)	18.3 (12.1)
Triplet	$(d_{x^2-y^2})^2(d_{xz})^2(d_{yz})^2(d_{z^2})^{\uparrow}(Cor)^{\uparrow}$	0.0 (0.0)	6.3 (-2.2)	9.6 (7.4)	NP

NP = not possible. [a] Values corrected for spin contamination, uncorrected values given within parentheses.

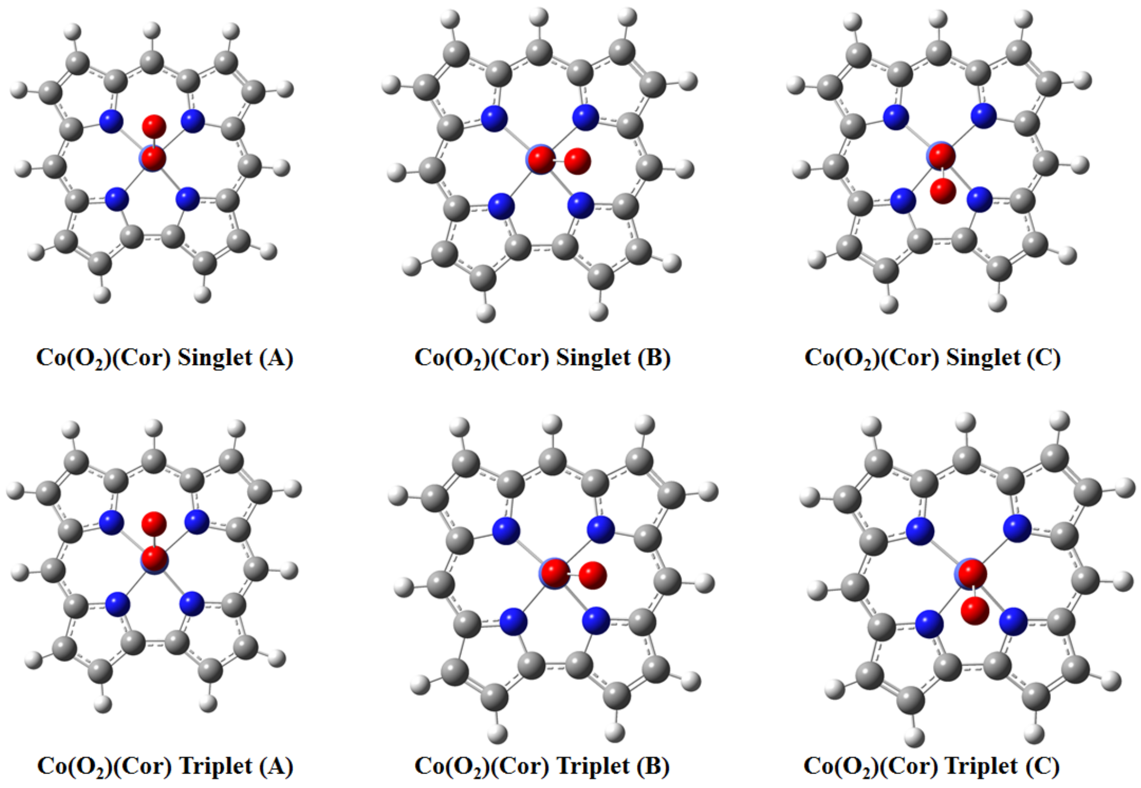

Figure S1. The optimized structures of Co(O_2)(Cor) obtained at the BP86/def2-TZVP level.

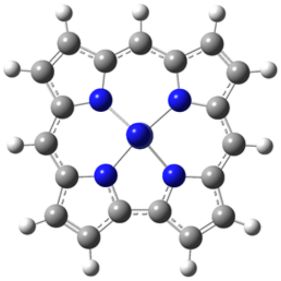

Co(N_2)(Cor) Singlet (A)

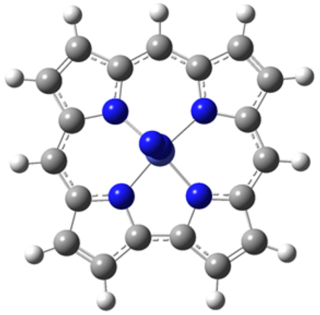

Co(N_2)(Cor) Triplet (A)

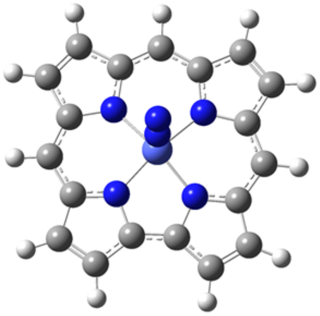

Co(N_2)(Cor) Triplet (B)

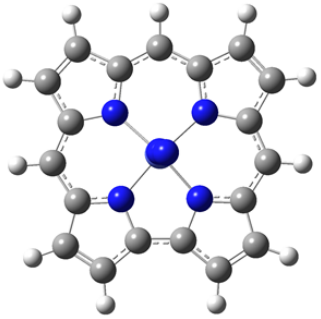

Co(N_2)(Cor) Triplet (C)

Figure S2. The optimized structures of Co(N_2)(Cor) obtained at the BP86/def2-TZVP level.

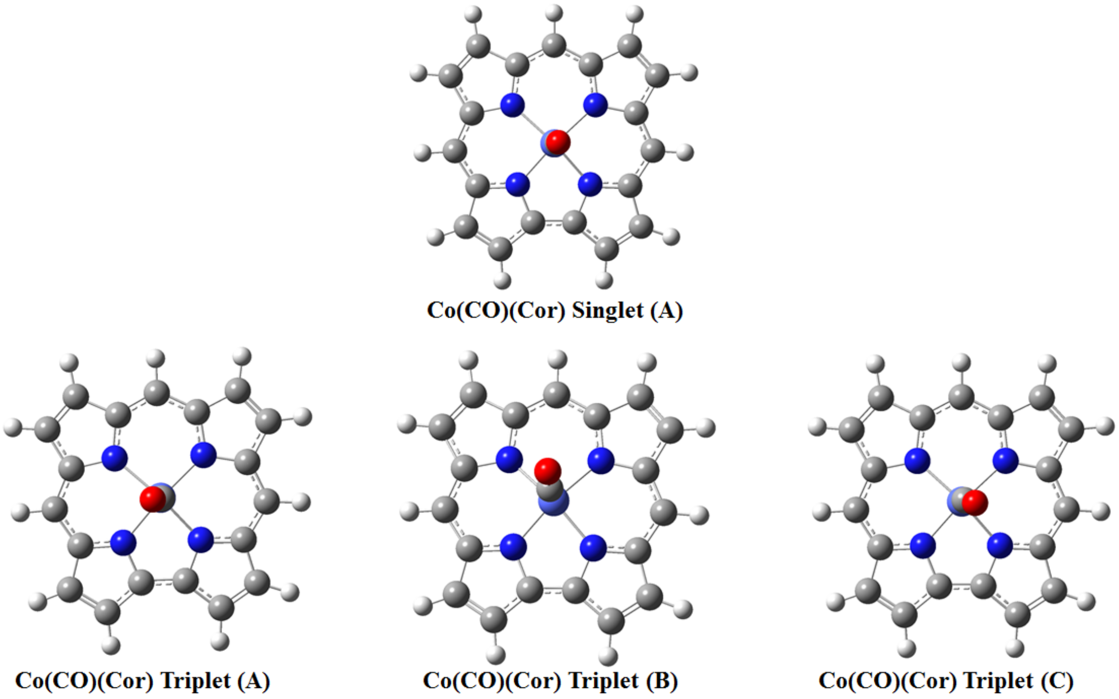

Figure S3. The optimized structures of Co(CO)(Cor) obtained at the BP86/def2-TZVP level.

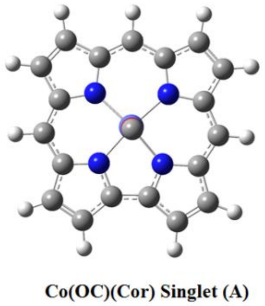

Figure S4. The optimized structure of Co(OC)(Cor) obtained at the BP86/def2-TZVP level.
